# Design of a Bovine Low-Density SNP Array Optimized for Imputation

**DOI:** 10.1371/journal.pone.0034130

**Published:** 2012-03-28

**Authors:** Didier Boichard, Hoyoung Chung, Romain Dassonneville, Xavier David, André Eggen, Sébastien Fritz, Kimberly J. Gietzen, Ben J. Hayes, Cynthia T. Lawley, Tad S. Sonstegard, Curtis P. Van Tassell, Paul M. VanRaden, Karine A. Viaud-Martinez, George R. Wiggans, for the Bovine LD Consortium

**Affiliations:** 1 UMR1313 Animal Genetics and Integrative Biology, National Institute for Agricultural Research (INRA), Jouy-en-Josas, France; 2 Animal Genetic Improvement Division, National Institute of Animal Science, Seonghwan, Cheonan, Republic of Korea; 3 Bovine Functional Genomics Laboratory, Agricultural Research Service, United States Department of Agriculture, Beltsville, Maryland, United States of America; 4 Institut de l'Elevage, Paris, France; 5 National Association of Livestock and Artificial Insemination Cooperatives (UNCEIA), Paris, France; 6 Illumina, San Diego, California, United States of America; 7 Biosciences Research Division, Department of Primary Industries Victoria, Melbourne, Victoria, Australia; 8 Dairy Futures Cooperative Research Centre, Bundoora, Victoria, Australia; 9 La Trobe University, Bundoora, Victoria, Australia; 10 Animal Improvement Programs Laboratory, Agricultural Research Service, United States Department of Agriculture, Beltsville, Maryland, United States of America; Auburn University, United States of America

## Abstract

The Illumina BovineLD BeadChip was designed to support imputation to higher density genotypes in dairy and beef breeds by including single-nucleotide polymorphisms (SNPs) that had a high minor allele frequency as well as uniform spacing across the genome except at the ends of the chromosome where densities were increased. The chip also includes SNPs on the Y chromosome and mitochondrial DNA loci that are useful for determining subspecies classification and certain paternal and maternal breed lineages. The total number of SNPs was 6,909. Accuracy of imputation to Illumina BovineSNP50 genotypes using the BovineLD chip was over 97% for most dairy and beef populations. The BovineLD imputations were about 3 percentage points more accurate than those from the Illumina GoldenGate Bovine3K BeadChip across multiple populations. The improvement was greatest when neither parent was genotyped. The minor allele frequencies were similar across taurine beef and dairy breeds as was the proportion of SNPs that were polymorphic. The new BovineLD chip should facilitate low-cost genomic selection in taurine beef and dairy cattle.

## Introduction

Genetic improvement of several key agricultural species is accelerating with the adoption of genomic selection [1], [2], [3]. With this method, animals or plants can be selected for breeding on the basis of their genetic merit predicted by markers spanning the entire genome. Particularly in dairy cattle, this method has been shown to be more efficient than conventional progeny testing of bulls (up to double the rate of genetic gain) as well as substantially less expensive [4]. Moreover, genomic selection opens new opportunities for sustainable management of populations by more efficiently selecting for traits that have low heritability, e.g. fitness traits, or traits that are difficult to measure. This method is also useful for managing the accumulation of inbreeding within breeds with a small effective population size. In dairy cattle, genomic selection has been deployed at a rapid pace, and most countries with major dairy breeding programs now rely heavily on this new technology [5].

A major challenge in implementing genomic selection in most species is the cost of genotyping. The expected value of the information gained by genotyping must exceed the cost of obtaining the genotypes. During the early stages of genomic selection in the dairy industry, the cost of high-density genotyping could be justified. The primary application was to evaluate bulls that were potential candidates for production of commercial semen. Using SNP information for those evaluations resulted in more accurate selection of bulls to acquire and extensively market. Once increased accuracies of genome-enhanced breeding values had been demonstrated, breeders and buyers quickly adopted this technology to improve accuracy of selection [6]. This example of a genomic-selection application has extreme value compared with other animal food production paradigms. In contrast, profit from genomic selection is likely to be much lower for beef bulls and dairy females [5], [7]. An appealing approach in situations with much lower returns from genotyping is to use a more economical, reduced-density SNP chip with markers optimized for imputation.

Imputation is the process of predicting unknown genotypes for animals from observed genotypes and often uses information from a reference population with dense genotypes to predict missing genotypes for animals with lower density genotypes. It is also applied to merge genotypes of similar densities but different SNPs. Most imputation algorithms use information from relatives and population linkage disequilibrium. A number of software programs for imputation have been developed based originally on human genetics [8], [9] and more recently on animal genetics [10], [11], [12], [13]. The limited effective population sizes and population structures in livestock allow the possibility of imputation of high-density genotypes from quite low-density genotypes [11], [14], [15], [16].

In 2010, a low-density bovine SNP chip, the Illumina GoldenGate Bovine3K Genotyping Beadchip (http://www.illumina.com/documents/products/datasheets/datasheet_bovine3K.pdf), was developed and made commercially available. That product offered a significant advance toward low-cost genomic selection in cattle; however, imputation accuracy was highly dependent on the relationship of the individual genotyped with the Bovine3K chip to the reference population genotyped at a higher density [17]. In addition, some samples failed to provide genotypes of adequate quality for use in genomic predictions. The SNP call rate performance of the Bovine3K chip was slightly reduced compared with the BovineSNP50 chip [18] because GoldenGate chemistry relies on two hybridization events for proper SNP detection as opposed to a single event for Infinium chemistry.

In this study, the Illumina Infinium BovineLD Genotyping Beadchip (http://www.illumina.com/documents/products/datasheets/datasheet_bovineLD.pdf) was developed to provide high imputation accuracy for higher density SNP genotypes in taurine dairy and beef populations. The main objective was to provide a tool that would enable genomic estimated breeding values to be calculated from accurately imputed genotype data from an Infinium-based SNP array with very low rates of failed samples. The main features of the new BovineLD chip are presented along with its imputation performance in a range of breeds and reference populations.

## Materials and Methods

### SNP selection

To provide highly accurate imputation to BovineSNP50 genotypes in global taurine breeds, SNPs were selected from validated assays from existing higher density chips and similar SNP detection technology, i.e. the Illumina BovineSNP50 and BovineHD (http://www.illumina.com/documents/products/datasheets/datasheet_bovineHD.pdf) SNP arrays, with priority given to BovineSNP50 content. From the known and validated SNPs, selection priority was 1) high minor allele frequencies (MAFs) in targeted breeds, 2) uniform spacing at a minimum of 2 SNPs per Mbp, with increased SNP density within 500 kbp of chromosomal ends, 3) inclusion of SNPs for determination of sex, parentage, Y haplotypes, and subspecies and maternal lineages, 4) SNP quality and fidelity criteria for robust reproducibility (>98% call rate and <0.01% Mendelian inconsistency), and 5) a target overlap of 2,000 SNPs with the Bovine3K chip to ensure backward compatibility. The anticipated SNP spacing (2 SNPs per Mbp) obviated the need to check for highly correlated SNPs.

The SNPs were selected to be highly informative with a high MAF over a large range of breeds from around the world (Table 1). The reference MAF estimates were from breeds in 10 countries from North America, Europe, and Oceania. Content selection was optimized using taurine allele frequencies. To achieve regular spacing, the UMD3 bovine genome assembly (http://www.cbcb.umd.edu/research/bos_taurus_assembly.shtml) was used to define 500-kbp segments over the 29 autosomes. A lack of flanking information at the end of each chromosome had resulted in lower imputation efficiency in preliminary tests. To correct that problem, the SNP density was doubled in the first and last segments of each chromosome. Reflecting the diverse membership of the Bovine LD Consortium, initial SNP selection was made by one member and updated by the others. The initial SNP selection was based on two independent criteria. First, SNPs with the highest mean MAF in each 500-kbp segment were selected over a broad range of European breeds including European Holstein, Montbéliarde, Normande, Jersey, Brown Swiss, Norwegian Red, Swedish Red and White, Finnish Ayrshire, Charolais, Limousine, Blonde d'Aquitaine, and Maine Anjou, with Holstein receiving double weight; the top two SNPs were selected in the segment at each end of the chromosome. Second, SNPs with the highest mean minimum MAF for six major European dairy breeds (European Holstein, Montbéliarde, Normande, Jersey, Brown Swiss, and Norwegian Red) were selected for each 500-kbp segment, with again 2 SNPs selected at each end of the chromosome. Selecting those SNPs with the highest mean of the two selection criteria within each 500-kbp segment (with doubling at the chromosome ends) resulted in 8,000 SNPs. Those 8,000 SNPs were subjected to a similar selection process using MAFs from North America and Oceania along with the European populations. For Holstein and Jersey breeds, the MAF used was the mean across the 3 populations; for Brown Swiss, only North America and Europe were included. The mean MAF was computed from Holstein, Jersey, Brown Swiss, Angus, and Brahman. The minimum MAF was from Jersey, Brown Swiss, and Angus. Again, the SNPs with the highest mean of the two selection criteria were selected with doubling at the chromosome ends.

**Table 1:** Number of DNA samples, minor allele frequencies (MAFs), and estimated frequency of loci that were polymorphic by breed and region.

			MAF		
Breed	Region	DNA samples (n)	Mean	Median	Loci that are polymorphic (%)
Angus	United States	6,400	0.33	0.35	98.3
	Australia	282	0.31	0.33	97.4
Ayrshire	North America	434	0.31	0.33	96.7
Beefmaster	United States	23	0.32	0.35	97.9
Blonde d'Acquitaine	Europe	160	0.34	0.37	98.5
Brahman	Australia	80	0.21	0.18	89.7
Brown Swiss	North America, Europe	2,039	0.31	0.34	96.2
Charolais	Europe	60	0.35	0.37	99.0
Fleckvieh	Europe	800	0.37	0.39	99.5
Friesian	New Zealand	17	0.35	0.38	98.8
Gelbvieh	North America	14	0.35	0.38	98.9
Guernsey	Global	61	0.29	0.30	93.2
Hereford	United States	24	0.31	0.33	96.1
Holstein	Australia	2,257	0.36	0.38	98.7
	North America	72,824	0.35	0.37	98.5
	Europe	16,000	0.36	0.38	98.9
Jersey	Australia	545	0.30	0.32	95.6
	North America	5,958	0.29	0.31	94.0
Limousin	Europe	90	0.35	0.37	98.4
Montbeliard	Europe	1,500	0.34	0.36	98.7
N'Dama	Africa	23	0.30	0.28	76.3
Normande	Europe	1,200	0.34	0.36	98.4
Norwegian Red	Norway	17	0.33	0.35	97.9
Red Angus	Angus	55	0.32	0.34	98.1
Red Danish	Europe	30	0.35	0.38	99.0
Santa Gertrudis	United States	21	0.32	0.33	97.2

Next, some of the selected SNPs were replaced by Bovine3K SNPs that were in nearby locations to ensure backward compatibility. In addition, SNPs used for breed determination and parentage testing that had not already been selected were included, and some SNPs were added to fill gaps generated by map inconsistencies.

For the X chromosome, Bovine3K SNPs with high MAFs were selected and supplemented with BovineSNP50 SNPs, with consideration given to spacing, MAF, and fidelity. Because large gaps remained after that initial selection, additional X- chromosome SNPs were chosen from the BovineHD assay.

For the Y chromosome and mitochondrial DNA (mtDNA), 9 Y-specific and 13 mtDNA SNP markers were identified from the BovineHD chip based on assay fidelity and performance across 27 breeds, MAF across those breeds, and ability of a SNP to discern subspecies and geographic locations of breed origins.

### Imputation

Imputation efficiency was assessed in 10 populations (North American, French, and Australian Holsteins; North American and Australian Jerseys; North American Brown Swiss; Australian Angus; French Montbéliarde; French Normande; and French Blonde d'Aquitaine). Beagle software (http://faculty.washington.edu/browning/beagle/beagle.html) [9] was used for the Australian and French populations and findhap.f90 (http://aipl.arsusda.gov/software/findhap/) [13] for the North American populations. These imputation programs have similar performance in large dairy cattle data sets [19]. Using existing genotypes from the BovineSNP50 chip, imputation efficiency was determined by comparing imputed and obseved genotypes. Part of the population was retained as a “reference,” while target individuals for imputation had their genotypes reduced in silico to either BovineLD or Bovine3K genotypes. Results were assessed as the proportion of genotypes that were correct in the target population. For example, if the imputed genotype was a heterozygote and the BovineSNP50 genotype was a homozygote, that genotype was counted as incorrectly imputed. The count of correct genotypes included both observed and imputed genotypes to measure the overall success of a lower density genotype in approximating a BovineSNP50 genotype.

### Content validation

The SNP assays for 6,914 loci were validated using data from 290 samples that represented 26 global dairy and beef breeds (Table 2) and included Bovine Hapmap samples [20]. The 290 samples (234 males, 56 females) included 286 unrelated samples, 2 trios, and 2 replicates. All markers were assessed for clustering of the genotypes using Illumina GenomeStudio genotyping software (version 2010.3; http://www.illumina.com/documents/products/datasheets/datasheet_genomestudio_software.pdf. A total of 6,909 clearly identifiable and scorable clusters were retained for robust utility of the panel. The cluster positions were defined with priority given first to data from dairy breeds and second to beef breeds. The purpose of the resulting cluster position file is to apply known robust cluster positions to future genotyping data for high throughput genotype calling. For phylogenetic analysis based on Y and mtDNA SNPs, individual sequences for each breed were clustered to construct consensus sequences using SNPs from 9 Y-chromosome loci and 13 mtDNA loci with the DNASTAR SeqMan program (version 6.1; http://www.dnastar.com/t-sub-products-lasergene-seqmanpro.aspx). There were 236 chromosome X SNP on the final Bovine LD chip. Flanking sequences and base calls for the 6,909 SNP are given in Table S1.

**Table 2:** Numbers of samples, call rates, and BovineSNP50 concordance for validation of BovineLD single-nucleotide polymorphisms (SNPs) by breed.

	Call rate		Concordance rate	
Breed	Samples (n)	Call rate (%)	Samples (n)	Concordance^a^ with BovineSNP50 SNPs (%)
Angus	10	99.98	10	99.997
Ayrshire	10	99.97	0	NA^b^
Beefmaster	10	99.85	10	99.974
Blonde d'Aquitaine	10	99.97	10	99.996
Brahman	10	99.5	10	99.972
Brown Swiss	10	100	10	99.999
Charolais	10	99.99	9	99.995
Fleckvieh	20	99.98	0	NA
Friesian	17	99.93	0	NA
Gelbvieh	5	99.97	0	NA
Guernsey	10	99.86	10	100
Hereford	10	99.86	10	99.997
Holstein	18	99.96	18	99.999
Jersey (United States)	19	99.96	19	100
Jersey (Denmark)	10	99.91	0	NA
Limousin	10	99.97	10	100
Montbeliard	10	100	9	99.995
N'Dama	10	99.85	10	100
Normande	10	99.98	10	99.997
Norwegian Red	11	99.88	11	100
Red Angus	10	99.99	10	100
Red Dairy (Angler)	10	99.99	0	NA
Red Danish (Denmark)	10	99.92	0	NA
Red Danish (Finland)	10	99.93	0	NA
Red Danish (Sweden)	10	99.84	0	NA
Santa Gertrudis	10	99.83	10	99.988
All breeds	290	99.93	186	99.995

^*a*^Concordance was included for animals with BovineSNP50 genotypes; “no calls” (null genotypes) on either BovineSNP50 or BovineLD were excluded from comparison.

^*b*^NA = not applicable.

## Results

### SNP call rates and accuracy

The BovineLD chip, consisting of 6,909 final loci, was validated for 290 individuals from 26 major dairy and beef breeds (Table 2). The mean call rate was 99.94% among dairy breeds, 99.90% among beef breeds, and 99.93% among all samples. For taurine breeds, discordant calls compared to BovineSNP50represented <0.01% of all genotyping calls (Table 2). Mendelian consistency was examined using two Holstein trios, which showed a single error on BTB-01149046 out of 13,797 total possible comparisons. Reproducibility was 100% across two Holstein replicated samples. Based on the nearly perfect concordance between the BovineLD and the BovineSNP50 genotypes reported in Table 2 and the similar concordance between BovineSNP50 and BovineHD genotypes, Mendelian consistency and reproducibility were also examined for the overlapping 6,844 SNPs from BovineHD genotypes. Those data included 8 parent-progeny, 24 parent-parent-progeny, and 10 replicate comparisons that represented 11 taurine, 2 indicine, and 1 hybrid breeds (Table 3). Mendelian consistency was 99.95%, and reproducibility was 99.99%.

**Table 3:** Mendelian consistency and reproducibility comparisons for a set of 6,844 SNPs in common for the BovineHD and BovineLD BeadChips.

						Correctly genotyped SNPs	
Statistic	Comparison	Breed	Comparisons (n)	SNPs genotyped (n)	Incorrectly genotyped SNPs (n)	(n)	(%)
Mendelian consistency	Parent-progeny pair	Angus	2	13,636	3	13,633	99.98
		Holstein	3	20,508	0	20,508	100
		Jersey	1	6,833	0	6,833	100
		N'Dama	1	6,720	0	6,720	100
		Red Angus	1	6,807	1	6,806	99.99
	Parent-parent-progeny trio	Angus	3	20,473	2	20,471	99.99
		Beefmaster	1	6,803	10	6,793	99.85
		Brahman	3	20,279	42	20,237	99.79
		Brown Swiss	2	13,597	0	13,597	100
		Charalois	3	20,325	7	20,318	99.97
		Hereford	2	13,607	3	13,604	99.98
		Holstein	4	27,283	2	27,281	99.99
		Jersey	3	20,438	5	20,433	99.98
		Santa Gertrudis	3	20,410	43	20,367	99.79
	Overall		32	217,719	118	217,601	99.95
Reproducibility	Replicates	Hereford	1	6,792	1	6,791	99.99
		Holstein	4	27,320	1	27,319	100
		Jersey	4	6,824	2	68,22	99.97
		Limousin	1	6,824	2	68,22	99.97
	Overall		10	47,760	6	47,754	99.99

The concordance rate for 2,088 SNPs in common between BovineLD and Bovine3K assays was 98.78% for 281 females genotyped with both chips. The most likely cause of the differential performance between the BovineLD and Bovine3K chips is the chemistry difference between the Infinium and GoldenGate assays.

### Performance for MAF, mean spacing, and paternal and maternal lineages

Data for calculating mean MAF (Table 1) were primarily BovineLD markers extracted from BovineSNP50 data. However, if BovineSNP50 data were not available, BovineLD markers from the validation data were used. That method allowed MAFs to be calculated more accurately. Mean MAF for the 6,909 SNPs was ≥0.29 for all taurine breeds (Table 1). For Brahman (a *Bos primigenius indicus* breed), mean MAF was lower (0.18). Overall, >89% of the SNPs were polymorphic in Brahman, which suggested that the BovineLD chip may be useful for imputation in this breed.

**Figure 1:**
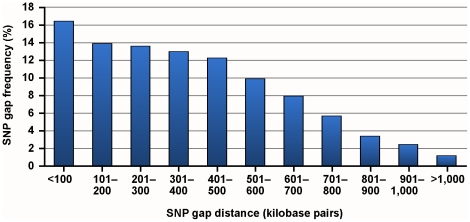
BovineLD single-nucleotide polymorphism (SNP) gap distribution.

**Figure 2:**
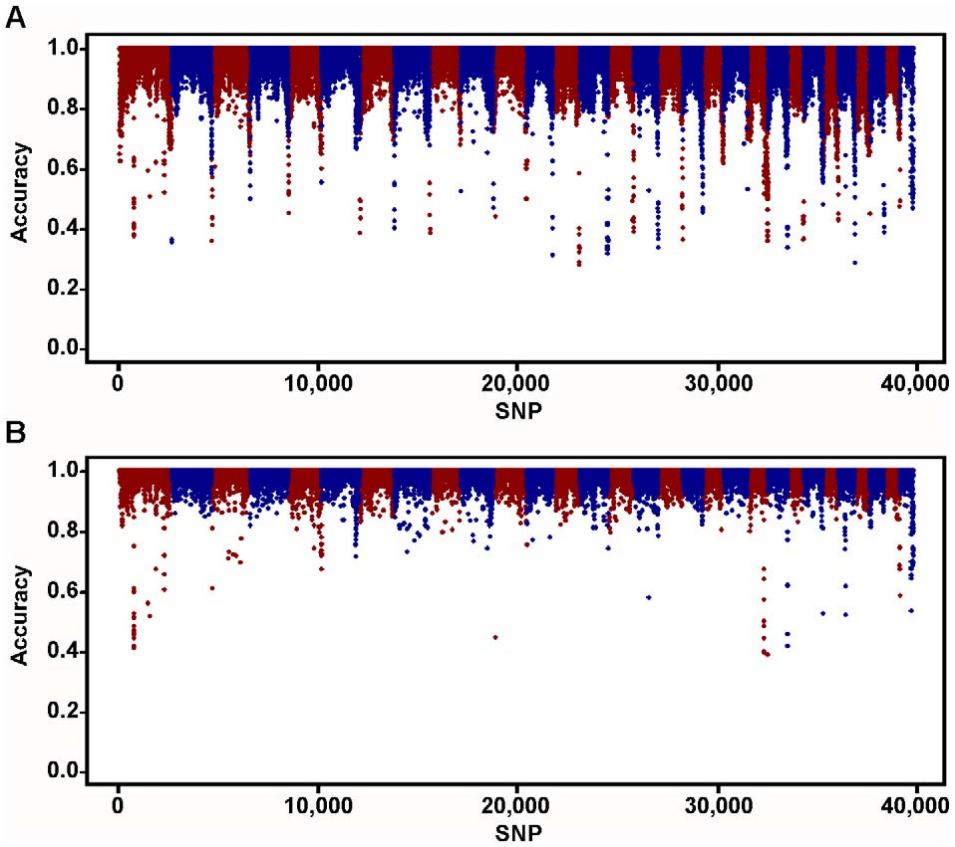
Imputation accuracy for Bovine3K and BovineLD genotypes. Imputation was performed for A) Bovine3K and B) BovineLD genotypes using Beagle software (http://faculty.washington.edu/browning/beagle/beagle.html); imputation accuracy is reported by single-nucleotide polymorphism (SNP).

For the 6,909 SNPs selected for the BovineLD chip, median spacing was 0.348 Mbp, with only 82 (1.1%) of intervals greater than 1 Mbp (Fig. 1). These gaps originate either from the X chromosome, or from regions not covered by the BovineSNP50. The strategy of increasing SNP density at chromosome ends substantially improved imputation accuracy for those regions compared with the Bovine3K array (Fig. 2).

The sex-specific and lineage identification SNPs also appeared to perform well. The nine Y-chromosome SNPs had a 100% call rate across 230 males of different breeds and no genotype calls for the 55 females. We investigated the frequency of the haplotypes of the alleles from these 9 SNP both within and across breeds. Four unique haplotypes were observed, which differed dramatically in frequency across breeds, Table 4. One haplotype, CGCCGCAAC (haplotype 1) was observed only in cattle with indicine lineage (eg Brahmans, Beef Master, Santa Getrudis). The second haplotype (TCTCCTCAC) was associated with central European lineage, haplotype 3 (TCTCCTCAT) was 1 base different from haplotype 2 and probably appeared to be associated with breeds that came to the island of Jersey from France or Spain, and haplotype 4 (TCTTGTCGC) was associated with northern European lineage, including islands. Only a few breeds had more than one haplotype, e.g. Santa Gertrudis and Beefmaster, both of which are taurine–indicine hybrids. Common haplotypes across breeds appeared to reflect a common origin. Phylogenetic analysis separated the 26 breeds into four distinctive clades, which agrees with a previous report on the dual origins of dairy cattle breeds in Europe [21]. For mtDNA SNPs (Table 5), seven unique mitochondrial haplotypes were found, however 259 of the animals sampled had the same mitochondrial haplotype. Haplotype 7 (AAGAGCAAAAAAG) was at highest frequency in indicine cattle. Most taurine×indicine cattle were derived from taurine cows. Therefore, the lack of haplotype 7 for taurine breeds in most regions is not unexpected. While more research is required, these preliminary results suggest the BovineLD markers could be useful in determining lineage origin between taurine and indicine breeds or identifying potential admixture within a population of locally adapted animals.

**Table 4:** Animal counts for Y-chromosome haplotypes^a^ by breed.

	Y-chromosome haplotype counts (n)			
Breed	1^b^	2^c^	3^d^	4^e^
Angus	0	0	0	9
Ayrshire	0	0	0	9
Beefmaster	2	0	0	5
Blonde d'Aquitaine	0	9	1	0
Brahman	7	0	0	3
Brown Swiss	0	10	0	0
Charolais	0	11	0	0
Fleckvieh	0	18	0	2
Friesian	0	0	5	12
Gelbvieh	0	4	0	1
Hereford	0	0	0	9
Holstein	0	0	0	15
Jersey	0	0	14	5
Limousin	0	10	0	0
Montbeliard	0	10	0	0
N'Dama	0	2	0	0
Normande	0	0	0	10
Norwegian Red	0	0	0	7
Red Angus	0	0	0	10
Red Dairy (Angler)	0	0	0	10
Red Danish	0	0	0	15
Santa Gertrudis	8	0	0	0
All breeds	17	74	20	122

^*a*^Haplotypes defined by SNP BovineHD310000-0048, -0099, -0103, -0210, -0515, -0517, -1188, -1404, and -1406.

^*b*^ CGCCGCAAC.

^*c*^ TCTCCTCAC.

^*d*^ TCTCCTCAT.

^*e*^ TCTTGTCGC.

**Table 5:** Animal counts for mtDNA-chromosome haplotypes^a^ by breed.

	mtDNA-chromosome haplotype counts (n)							
Breed	1^b^	2^c^	3^d^	4^e^	5^f^	6^g^	7^h^	Could not be determined
Angus	10	0	0	0	0	0	0	0
Ayrshire	9	0	0	0	1	0	0	0
Beefmaster	7	0	0	0	1	0	0	1
Blonde d'Aquitaine	10	0	0	0	0	0	0	0
Brahman	8	0	0	0	0	0	3	0
Brown Swiss	9	0	0	1	0	0	0	0
Charolais	10	0	0	0	0	0	0	0
Fleckvieh	20	0	0	0	0	0	0	0
Friesian	16	0	0	0	0	0	1	0
Gelbvieh	3	0	0	0	0	0	0	2
Guernsey	10	0	0	0	0	0	0	0
Hereford	9	0	0	0	0	0	1	0
Holstein	16	0	0	0	0	0	2	0
Jersey	21	0	0	0	0	1	5	1
Limousin	10	0	0	0	0	0	0	0
Montbeliard	10	0	0	0	0	0	0	0
N'Dama	10	0	0	0	0	0	0	0
Normande	9	0	0	0	1	0	0	0
Norwegian Red	6	0	0	0	1	0	0	4
Red Angus	9	1	0	0	0	0	0	0
Red Dairy (Angler)	10	0	0	0	0	0	0	0
Red Danish	28	0	1	1	1	0	0	0
Santa Gertrudis	9	0	0	0	1	0	0	0
All breeds	259	1	1	2	6	1	12	8

^*a*^Haplotypes defined by SNP BovineHD320000-0141, -0145, -0180, -0226, -0252, -0312, -0332, -0342, -0354, -0358, -0368, -0384, and -0406.

^*b*^ CCGCAACCGCCCG.

^*c*^ CCGCAAACGCCCG.

^*d*^ CCGCAACAGCCCG.

^*e*^ CCGCAACCACCCG.

^*f*^ CCGCAACCGCCCA.

^*g*^ CAACAACCGCCCG.

^*h*^ AAGAGCAAAAAAG.

### Accuracy of imputation

Imputation accuracy was assessed in Australian, French, and North American cattle populations. In all cases, the accuracy of imputation to BovineSNP50 genotypes was ≥95% (Table 6). Most imputation results were >97%, particularly for dairy breeds. The results were lower for some breeds, likely because of the limited reference population size used. For example, the considerably larger size of the North American reference set of Holsteins compared with the Australian set could explain why the North American imputation accuracy was 1.1 percentage points higher than for Australia. The effect of a smaller reference set of genotypes on imputation accuracy was further demonstrated by imputation from BovineLD genotypes for Australian Angus, which had the smallest reference population in the data set. For French populations, imputation efficiency also varied, with the highest accuracy for Holsteins and the lowest for Blondes d'Aquitaine (Table 6); imputation accuracy for Normandes and Montbéliardes was slightly lower than for Holsteins. Again, much of the variation is likely explained by reference population size.

**Table 6:** Accuracy of imputation from BovineLD genotypes to BovineSNP50 genotypes for Australian, French, and North American breeds.

				Imputation accuracy	
Country/region^a^	Breed	Reference	Target	Genotypes correctly imputed (%)^b^	Known genotypes without error (%)^c^
Australia	Angus	200	82	92.3	93.1
	Holstein	1,831	360	97.5	97.8
	Jersey	454	86	94.9	95.7
France	Blonde d'Aquitaine	753	237	95.2	95.8
	Holstein	3,505	966	98.5	98.7
	Montbéliarde	1,170	222	98.1	98.4
	Normande	1,176	248	98.4	98.6
North America	Brown Swiss	1,994	168	97.4	97.9
	Holstein	63,288	19,506	98.8	98.9
	Jersey	8,687	1,140	98.0	98.3

^*a*^Beagle software (http://faculty.washington.edu/browning/beagle/beagle.html) was used for Australian and French imputations and findhap.f90 (http://aipl.arsusda.gov/software/findhap/) for North American imputations.

^*b*^The 6,909 SNPs on the BovineLD chip were excluded from the calculation of imputation accuracy.

^*c*^All SNPs included, i.e. the 6,909 SNPs on the BovineLD chip.

For Australian and North American Holsteins, accuracy of imputation to BovineSNP50 genotypes was better for BovineLD genotypes than for Bovine3K genotypes. For Australian Holsteins, imputation accuracies were up to almost 6 percentage points higher with the BovineLD chip than with the Bovine3K chip using the same data (Table 7). Mean imputation accuracy was 92.8% for Australian Holstein Bovine3K genotypes compared with 97.6% for BovineLD genotypes. For North American Holsteins, accuracies of imputation to BovineSNP50 genotypes from Bovine3K genotypes ranged from 93.0 to 96.7% (depending on number of parents genotyped) for 2,456 animals genotyped with both Bovine3K and BovineSNP50 chips [17]. Corresponding values for BovineLD genotypes (Table 8) are 96.6 to 99.3%.

**Table 7:** Accuracy of imputation^a^ from BovineLD or Bovine3K genotypes to BovineSNP50 genotypes for Australian Holsteins with and without a sire in the reference population^b^.

Sire status	Genotyping chip	Animals imputed (n)	Imputation accuracy (%)
Included in reference population	BovineLD	240	98.3
	Bovine3K	240	94.2
Not included in reference population	BovineLD	120	97.0
	Bovine3K	120	91.3

^*a*^Imputation was done using Beagle software (http://faculty.washington.edu/browning/beagle/beagle.html).

^*b*^Reference population included 1,831 animals.

**Table 8:** Accuracy of imputation^a^ from BovineLD genotypes to BovineSNP50 genotypes for North American Brown Swiss, Holsteins, and Jerseys with and without parents in the reference population^b^.

		Jersey		Holstein		Brown Swiss	
Sire genotype	Dam genotype	Animals with imputed genotypes (n)	Genotypes imputed correctly (%)	Animals with imputed genotypes (n)	Genotypes imputed correctly (%)	Animals with imputed genotypes (n)	Genotypes imputed correctly (%)
BovineSNP50	BovineSNP50	345	99.1	9,319	99.3	13	99.0
BovineSNP50	None	593	98.1	9,383	98.7	145	97.9
None	BovineSNP50	6	98.1	135	98.5	1	97.2
BovineSNP50	Bovine3K	158	98.3	158	98.8	NA^c^	NA
Bovine3K	None	3	96.9	NA	NA	NA	NA
None	Bovine3K	1	96.6	8	97.8	NA	NA
None	None	34	92.7	389	96.6	9	95.1
All comparisons		1,140	98.3	19,506	98.9	168	97.9

^*a*^Imputation was done using findhap.f90 (http://aipl.arsusda.gov/software/findhap/), which includes both population- and pedigree-based haplotypes.

^*b*^Reference population included 63,288 animals.

^*c*^NA = not applicable.

The greatest improvement in imputation for BovineLD genotypes compared with Bovine3K genotypes was for individuals with no genotyped parents. For Australian Holsteins, difference in mean imputation accuracy with and without a sire in the reference population was 2.9 percentage points for Bovine3K genotypes but only 1.3 percentage points for BovineLD genotypes. The improvement was smaller for North American Holsteins: a difference of 2.7 percentage points between both parents genotyped and no genotyped parents for Bovine LD genotypes (Table 6) compared with 3.7% for Bovine3K genotypes [17]. Compared with North American Holsteins, BovineLD imputation accuracy for animals without a parent in the reference population was slightly poorer for North American Jersey and Brown Swiss populations (Table 8). However, the more than doubling of markers and the different SNP selection criteria [22] compared with the Bovine3K chip allowed high imputation accuracies across a wider range of dairy breeds as well as some beef breeds.

## Discussion

The Illumina BovineLD BeadChip includes 6,909 SNPs selected to provide optimized imputation to BovineSNP50 genotypes in dairy breeds. The SNPs have MAFs of >0.3 in most breeds, and nearly uniform spacing across the genome except at the ends of the chromosome where densities were increased. The chip also includes SNPs on the Y chromosome and mtDNA loci that are useful for gender checking, determining subspecies classification and identifying certain paternal and maternal breed lineages. Accuracy of imputation to BovineSNP50 genotypes using the BovineLD chip was >99% when both parents were genotyped in the North American BovineSNP50 reference population. That high accuracy suggests that the design criteria for the BovineLD chip would be useful to consider in other species for which an “imputation chip” could dramatically lower the cost of implementing genomic selection. BovineLD imputation was about 3 percentage points more accurate across multiple populations compared with Bovine3K imputation. The improvement was greatest when neither parent had been genotyped. The gain in imputation accuracy is attributed primarily to the increased overall density of the BovineLD chip compared with the Bovine3K chip and also to the even further increased density at the ends of chromosomes. The high MAFs also contribute to the improved imputation accuracy. The MAFs were similar across taurine beef and dairy breed as was the proportion of SNPs that were polymorphic. Although it would be expected that accuracies of imputation would be highest for those breeds which were included in the design of the chip, which was dominated by dairy breeds, the similar SNP characteristics (particularly the high MAF across many beef and dairy taurine breeds) suggest that the BovineLD chip will perform well in imputation of taurine beef cattle. Our results suggest that the imputation accuracy will also be quite dependent on the size of the population genotyped with a higher density SNP assay. Overall, the new BovineLD BeadChip should facilitate low cost genomic selection in *Bos primigenius taurus* beef and dairy cattle.

## Supporting Information

Table S1 Genomic locations, flanking sequences and base calls for the 6,909 SNP on the bovineLD array.
(CSV)

## References

[1] Meuwissen THE, Hayes BJ, Goddard ME (2001). Prediction of total genetic value using genome-wide dense marker maps.. Genetics.

[2] Heffner EL, Jannink JL, Sorrells ME (2011). Genomic selection accuracy using multifamily prediction models in a wheat breeding program.. The Plant Genome.

[3] Wiggans GR, VanRaden PM, Cooper TA (2011). The genomic evaluation system in the United States: past, present, future.. J Dairy Sci.

[4] Schaeffer LR (2006). Strategy for applying genome-wide selection in dairy cattle.. J Anim Breed Genet.

[5] Pryce JE, Goddard ME, Raadsma HW, Hayes BJ (2010). Deterministic models of breeding scheme designs that incorporate genomic selection.. J Dairy Sci.

[6] Pryce JE, Daetwyler HD (2011). Designing dairy cattle breeding schemes under genomic selection—a review of international research.. Anim Prod Sci.

[7] Van Eenennaam AL, van der Werf JHJ, Goddard ME (2011). The value of using DNA markers for beef bull selection in the seedstock sector.. J Anim Sci.

[8] Scheet P, Stephens M (2006). A fast and flexible statistical model for large-scale population genotype data: applications to inferring missing genotypes and haplotypic phase.. Am J Hum Genet.

[9] Browning SR, Browning BL (2011). Haplotype phasing: existing methods and new developments.. Nat Rev Genet.

[10] Druet T, Georges M (2010). A hidden Markov model combining linkage and linkage disequilibrium information for haplotype reconstruction and quantitative trait locus fine mapping.. Genetics.

[11] Daetwyler HD, Wiggans GR, Hayes BJ, Woolliams JA, Goddard ME (2011). Imputation of missing genotypes from sparse to high density using long-range phasing.. Genetics.

[12] Hickey JM, Kinghorn BP, Tier B, Wilson JF, Dunstan N (2011). A combined long-range phasing and long haplotype imputation method to impute phase for SNP genotypes.. Genet Sel Evol.

[13] VanRaden PM, O'Connell JR, Wiggans GR, Weigel KA (2011). Genomic evaluations with many more genotypes.. Genet Sel Evol.

[14] Druet T, Schrooten C, de Roos AP (2010). Imputation of genotypes from different single nucleotide polymorphism panels in dairy cattle.. J Dairy Sci.

[15] Weigel KA, Van Tassell CP, O'Connell JR, VanRaden PM, Wiggans GR (2010). Prediction of unobserved single nucleotide polymorphism genotypes of Jersey cattle using reference panels and population-based imputation algorithms.. J Dairy Sci.

[16] Dassonneville R, Brøndum RF, Druet T, Fritz S, Guillaume F (2011). Effect of imputing markers from a low-density chip on the reliability of genomic breeding values in Holstein populations.. J Dairy Sci.

[17] Wiggans GR, Cooper TA, VanRaden PM, Olson KM, Tooker ME (2012). Use of the Illumina Bovine3K BeadChip in dairy genomic evaluation.. J Dairy Sci.

[18] Matukumalli LK, Lawley CT, Schnabel RD, Taylor JF, Allan MF (2009). Development and characterization of a high density SNP genotyping assay for cattle.. PLoS One.

[19] Johnston J, Kistemaker G, Sullivan PG (2011). Comparison of different imputation methods. Interbull Bulletin 44.. http://www.interbull.org/images/stories/Jarmila_copy.pdf.

[20] The Bovine Hap Map Consortium (2009). Genome-wide survey of SNP variation uncovers the genetic structure of cattle breeds.. Science.

[21] Edwards CJ, Ginja C, Kantanen J, Pérez-Pardal L, Tresset A (2011). Dual origins of dairy cattle farming—evidence from a comprehensive survey of European Y-chromosomal variation.. PLoS ONE.

[22] Dassonneville R, Fritz S, Ducrocq V, Boichard D (2012). Short Communication: Imputation performances of three low density marker panels in beef and dairy cattle.. J Dairy Sci.

